# Hypoxia-responsive miR-210 promotes self-renewal capacity of colon tumor-initiating cells by repressing ISCU and by inducing lactate production

**DOI:** 10.18632/oncotarget.11772

**Published:** 2016-08-31

**Authors:** Pit Ullmann, Komal Qureshi-Baig, Fabien Rodriguez, Aurélien Ginolhac, Yannic Nonnenmacher, Dominik Ternes, Jil Weiler, Karoline Gäbler, Christelle Bahlawane, Karsten Hiller, Serge Haan, Elisabeth Letellier

**Affiliations:** ^1^ Life Sciences Research Unit, University of Luxembourg, L-4367 Belvaux, Luxembourg; ^2^ Luxembourg Centre for Systems Biomedicine, L-4367 Belvaux, Luxembourg

**Keywords:** colorectal cancer, tumor-initiating cell, hypoxia, miR-210, self-renewal capacity

## Abstract

Low oxygen concentrations (hypoxia) are known to affect the cellular metabolism and have been suggested to regulate a subpopulation of cancer cells with tumorigenic properties, the so-called tumor-initiating cells (TICs). To better understand the mechanism of hypoxia-induced TIC activation, we set out to study the role of hypoxia-responsive miRNAs in recently established colon cancer patient-derived TICs. We were able to show that low oxygen concentrations consistently lead to the upregulation of miR-210 in different primary TIC-enriched cultures. Both stable overexpression of miR-210 and knockdown of its target gene *ISCU* resulted in enhanced TIC self-renewal. We could validate the tumorigenic properties of miR- 210 in *in vivo* experiments by showing that ectopic expression of miR-210 results in increased tumor incidence. Furthermore, enhanced miR-210 expression correlated with reduced TCA cycle activity and increased lactate levels. Importantly, by blocking lactate production via inhibition of LDHA, we could reverse the promoting effect of miR-210 on self-renewal capacity, thereby emphasizing the regulatory impact of the glycolytic phenotype on colon TIC properties. Finally, by assessing expression levels in patient tissue, we could demonstrate the clinical relevance of the miR-210/ISCU signaling axis for colorectal carcinoma. Taken together, our study highlights the importance of hypoxia-induced miR-210 in the regulation of colon cancer initiation.

## INTRODUCTION

Colorectal cancer (CRC) is among the most prevalent cancers worldwide with more than 1.3 million diagnoses and almost 700,000 deaths per year [1]. Cancer patient survival is largely dependent on early diagnosis and intervention. Up to 30% of stage II patients relapse after surgery and many of them die due to metastatic disease [2]. Accordingly, there is a great need for the identification of new biomarkers and for a better understanding of CRC initiation.

Tumor-initiating cells (TICs), also known as cancer stem cells (CSCs), have been identified as a subgroup of cells that are able to drive initiation, maintenance and progression of CRC [3, 4]. A deeper insight into the regulation of colon TICs might thus favor the development of more effective CRC prevention and therapy. Only a limited number of cells within CRC tissue as well as within conventional cancer cell lines displays TIC properties [4]. Most data generated in cell lines are therefore based on the behavior of rather differentiated cells whereas the response of TICs remains majorly unknown. Studies on TIC biology have been limited so far, in part due to intense controversy regarding the use of surface markers for their isolation and characterization [5–7]. To address these issues, we have recently established and characterized different CRC spheroid cultures (SCs), both from patient samples and from conventional CRC cell lines, by rather relying on functional properties than on surface marker expression. We could show that these SCs retain most important characteristics of their tumor of origin and display pronounced TIC features, such as self-renewal capacity, tumorigenic potential, and chemoresistance [8].

Due to excessive proliferation, irregular blood flow and abnormal neovascularization, the interior of solid tumors is progressively exposed to reduced oxygen levels [9]. Several studies have shown that hypoxia contributes to a more aggressive cancer phenotype by driving invasion, metastasis, resistance to conventional therapies, as well as tumor recurrence [10]. Accordingly, it has been suggested that hypoxia signaling correlates with poor cancer patient prognosis [11]. Besides, hypoxic regions within tumors have been found to overlap with TIC niches [12] and emerging evidence indicates that hypoxia is determinant in maintaining the stem-like fraction of tumor cells in different cancer types [13–16], including CRC [17].

By inducing the production of reactive oxygen species, by interfering with oxidative phosphorylation, and by promoting a glycolytic phenotype, hypoxia is also known to have a huge impact on cancer cell metabolism [18]. Moreover, it has been shown that hypoxia-inducible factor 1α (HIF-1α) is sufficient to press human embryonic stem cells to switch their metabolism from bivalent energy production to a mainly glycolytic phenotype [19]. In addition, pluripotent cells are thought to be more glycolytic than fully differentiated somatic cells [20], thus it seems reasonable to assume that hypoxia-induced metabolic reprogramming also affects the TIC pool within a tumor. Nonetheless, the exact molecular mechanisms through which reduced oxygen levels influence TIC behavior remain poorly understood.

Several lines of evidence now postulate miRNAs, which are short non-coding RNAs modulating gene expression through post-transcriptional regulation, as key elements in the hypoxic response [21]. In particular, miR-210, often referred to as master hypoxamiR, has been identified as the most prominent and consistently up-regulated miRNA under hypoxic conditions [22]. Depending on the cellular context, miR-210 can act both as tumor suppressor by reducing cell proliferation and as oncogene by regulating apoptosis, invasion, and cellular metabolism (reviewed in [23]). Besides, miR-210 has been shown to indirectly promote tumor growth by enhancing myeloid-derived suppressor cell-mediated T-cell suppression and by repressing the susceptibility to cytotoxic T lymphocyte-driven lysis of tumor cells [24, 25]. Recently, the clinical relevance of miR-210 has been evaluated and high miR-210-3p expression has been associated with poor patient prognosis in several epithelial cancers [26, 27]. Thus, hypoxia-responsive miRNAs (HRMs), such as miR-210, may represent interesting CRC biomarkers as well as target molecules for gene and drug therapy. However, the role of miR-210 in the regulation of TICs and CRC tumorigenesis remains largely unknown. Accordingly, in the present study, we strive to investigate how reduced oxygen levels and hypoxia-responsive miR-210 affect colon TIC behavior and thereby drive tumorigenesis.

## RESULTS

### Hypoxia promotes self-renewal of colon TICs

**Figure 1:**
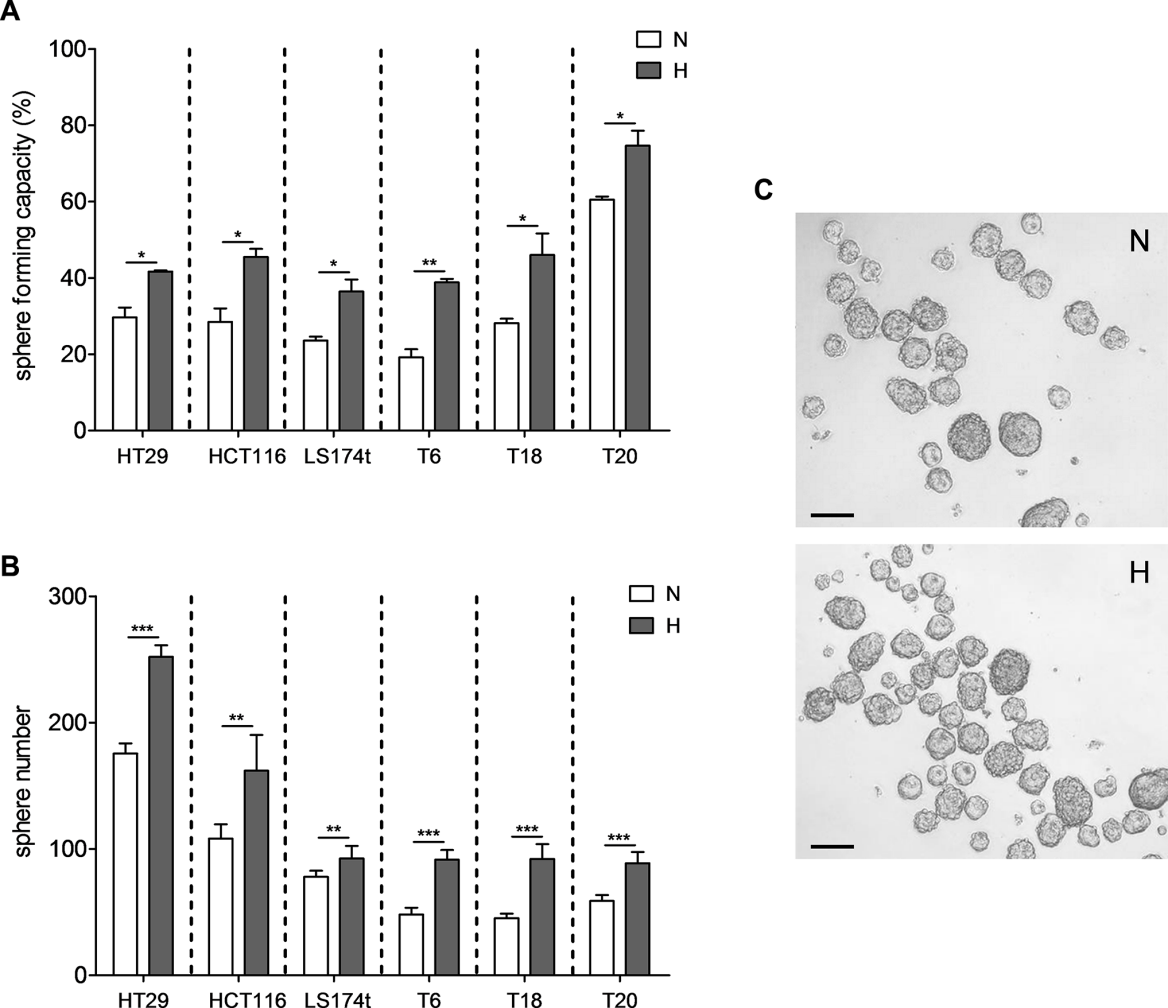
Hypoxia promotes self-renewal capacity of colon TICs Sphere formation assays were performed for SCs derived from HT29, HCT116 and LS174t CRC cell lines as well as for SCs generated from primary colon tumors T6, T18 and T20. (**A**) Single cells or (**B**) 1,000 cells were seeded and maintained under normoxic or hypoxic conditions and spheroids were counted after 10 or 7 days, respectively. (**C**) Representative image of a T20 1,000 cell assay after 7 days under normoxic or hypoxic conditions; scale bar corresponds to 100 μm. Representative figure of at least 3 independent experiments for (A) and (B). Data are presented as mean ± SD. Unpaired Student's *t*-test was used to compare both conditions; *p < 0.05, **p < 0.01, and ***p < 0.001; N–normoxia, H–hypoxia.

We first investigated the effect of hypoxia on the growth behavior of TICs by assessing the self-renewal capacity of three primary established (T6, T18 and T20) as well as three cell line-derived (HT29, HCT116 and LS174t) SCs at 1% oxygen levels. These SCs have previously been fully characterized and were shown to be enriched in TICs [8]. Sphere formation assays, performed at single cell and 1,000 cells/well densities, showed that self-renewal capacity of all different SCs was increased under hypoxic conditions (Figure 1). The hypoxia-mediated increase in sphere formation was thereby maintained over several passages (Supplementary Figure S1), supporting the hypothesis that low O_2_ levels stably promote self-renewal of primary and cell line-derived TICs.

### Hypoxia induces upregulation of miR-210 in colon TICs

**Figure 2:**
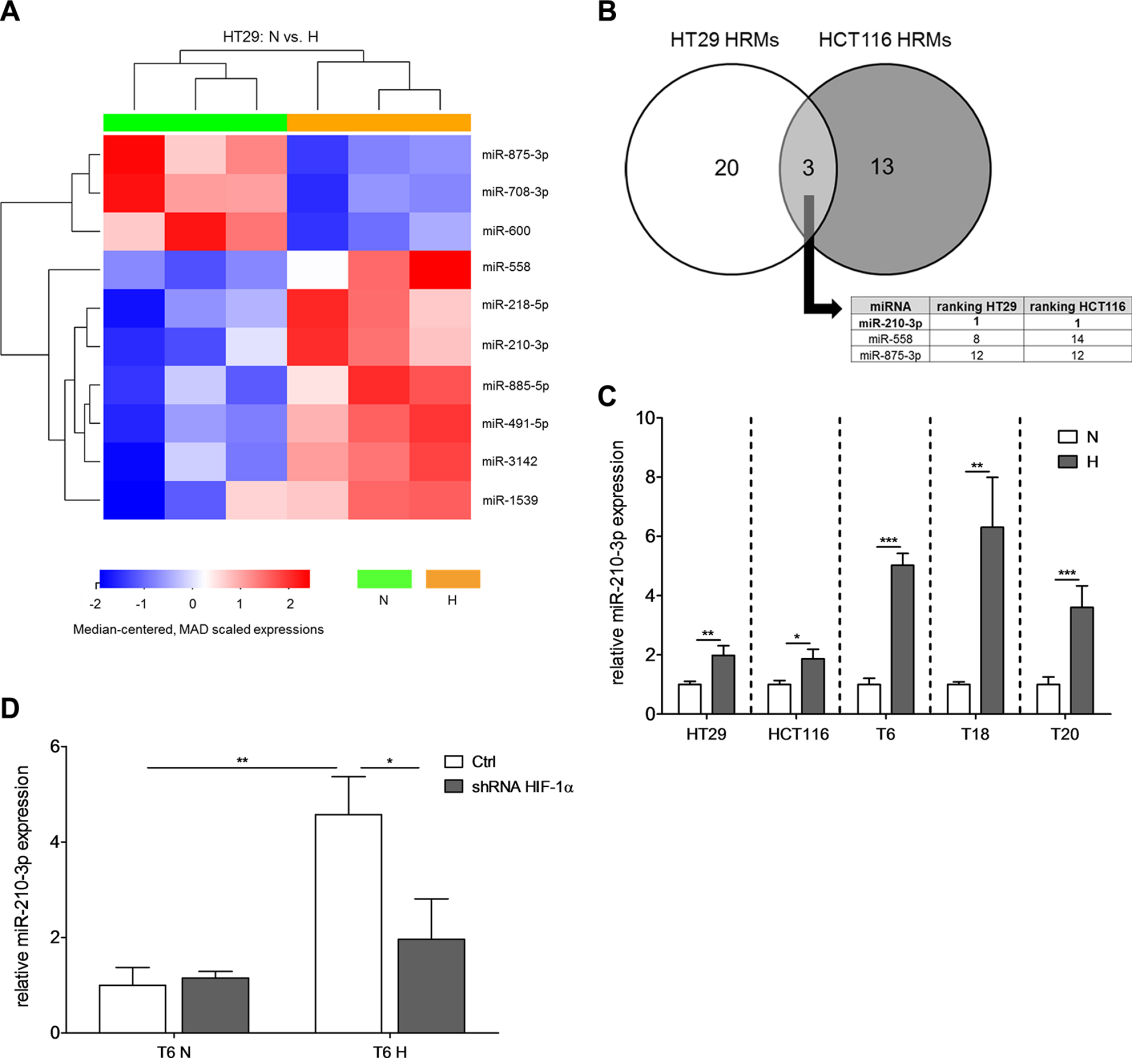
miR-210 is commonly upregulated in different SCs under hypoxic conditions (**A**) Heatmap depicting the 10 most significantly (according to *p*-value) deregulated miRNAs for HT29 SCs after 72 h of hypoxic culture conditions. miRNA expression levels of both normoxia and hypoxia cultures were measured with WaferGen SmartChip Human miRNA Panel v3.0 qPCR arrays. (**B**) Determination of commonly deregulated miRNAs among HT29 and HCT116 SCs. In the table insert, hypoxia-responsive miRNAs (HRMs) were ranked according to their absolute fold change values. (**C**) Relative miR-210-3p expression of different SCs after 72 h hypoxia; representative figure of at least 3 independent experiments. (**D**) Relative expression of miR-210-3p after stable knockdown of HIF-1α after 72 h under normoxic or hypoxic conditions, respectively; representative figure of 2 independent experiments. Data are presented as mean ± SD and unpaired Student's *t*-tests were used to test for statistical significance in (C) and (D); *p < 0.05, **p < 0.01, and ***p < 0.001; N–normoxia, H–hypoxia, shRNA–short hairpin RNA.

MicroRNAs have recently been identified as major regulators of the hypoxic response [21]. In order to identify hypoxia-responsive miRNAs (HRMs) in CRC, we performed WaferGen SmartChip qPCR-based arrays, which allow the simultaneous probing of 1,036 miRNAs. Using two of our characterized TIC cultures, miR-210 was identified as the miRNA with the strongest response to hypoxia in both SCs (Figure 2A–2B, Supplementary Table S1). These results were validated by assessing the expression of miR-210-3p in all our different SCs. After 72 h at 1% O_2_, miR-210-3p expression was increased by 2- to 6-fold (Figure 2C). Interestingly, the hypoxia-induced upregulation of miR-210 was thereby stronger in primary than in cell line-derived TICs (Figure 2C). In agreement with others [28–30], we could show that miR- 210 is regulated in a HIF-1α-dependent manner, as stable knockdown of HIF-1α reversed the upregulation of miR- 210 under hypoxic conditions (Figure 2D, Supplementary Figure S2A for knockdown efficiency).

### miR-210 promotes self-renewal capacity of colon TICs

**Figure 3:**
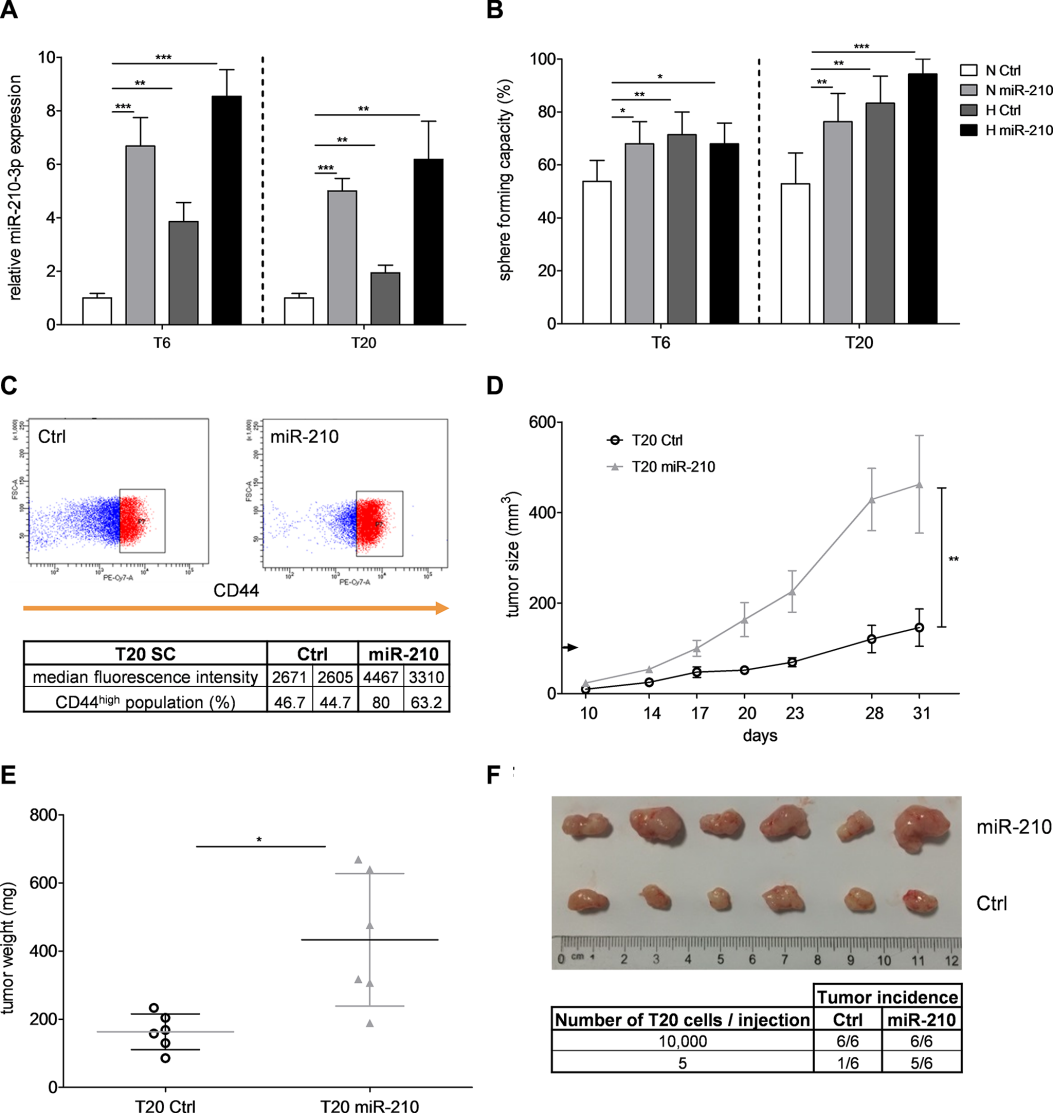
miR-210 enhances self-renewal capacity and tumorigenic potential of colon TICs (**A**) Relative expression of miR-210-3p after 72 h under normoxia or hypoxia was assessed by qPCR, after lentiviral transduction of miR-210 or control vectors in T6- and T20-derived SCs. Representative figure of 3 independent experiments; data are presented as mean ± SD. Unpaired Student's *t*-test was used to compare two groups. (**B**) Self-renewal capacity, determined with limiting dilution assays, after stable overexpression of miR-210 in T6 and T20 SCs. Representative figure of 3 independent experiments; data presented as mean with 95% confidence interval; statistical significance was assessed with a Chi-square test using the ELDA software. (**C**) CD44 expression in T20 SCs after stable transduction of miR-210 or control vector. Lower panel comprises median fluorescence intensity values and percentage of CD44^high^ population of 2 independent experiments. (**D**) *In vivo* tumor growth after subcutaneous injection of 10,000 T20 cells with/without stable overexpression of miR-210. Data are presented as mean ± SEM; two-way ANOVA was used to test for statistical significance; *n* = 6 mice/group. (**E**) T20 tumor weight after overexpression of miR-210. Data are presented as mean ± SD; paired Student's *t*-test was used to test for statistical significance; *n* = 6 mice/group. (**F**) Extracted T20 tumors after injection of 10,000 cells, following stable transduction of miR-210 or respective control vector. Table in the lower panel shows effect of miR-210 on tumor incidence with different cell numbers per injection. *p < 0.05, **p < 0.01, and ***p < 0.001; N–normoxia, H–hypoxia.

Next, we investigated whether miR-210 is directly involved in the hypoxia-induced TIC behavior. Stable overexpression of miR-210 led to a 3- to 7-fold upregulation of miR-210-3p expression in our primary SCs (Figure 3A). Unlike miRNA mimic transfection, which yielded very high expression levels (data not shown), stable overexpression via lentiviral transduction allowed us to obtain more physiological levels of miR- 210-3p (Figure 3A). Importantly, overexpression of miR-210 under normoxia raised the sphere formation capacity of TICs to a similar level as hypoxia (Figure 3B and Supplementary Figure S3A), suggesting that miR- 210 is driving the hypoxia-mediated increase in TIC self-renewal activity. Despite remaining discord regarding the use of surface markers for the identification and isolation of TICs [6], CD44 has recently emerged as a potential TIC marker, promising biomarker candidate and therapeutic target [31, 32], especially in the field of colon TICs [33]. Interestingly, we could observe an increased expression of CD44 after lentiviral transduction of miR-210 in T20 SCs (Figure 3C), further hinting at a potential link between miR-210 and colon TIC regulation. Besides, clonogenic capacity of primary SCs was increased after overexpression of miR-210, although the effect was smaller on colony than on sphere formation (Supplementary Figure S3B). On the other hand, sphere size, proliferation, cell viability and apoptotic rates remained unaffected (Supplementary Figure S3C– S3D and S3F–S3G), suggesting that miR-210 mainly regulates TIC self-renewal, rather than proliferation. In order to investigate the role of miR-210 in *in vivo* tumor initiation, we analyzed the tumorigenic capacity of cells stably transduced with miR-210. Overexpression of miR- 210 resulted in significantly increased tumor growth (Figure 3D), weight (Figure 3E) and size (Figure 3F) compared to the respective control groups. Importantly, tumor incidence following injections of low cell numbers was higher after stable overexpression of miR-210 (Figure 3F), emphasizing that miR-210 regulates tumor initiation. Levels of miR- 210- 3p remained high in extracted T20 tumors, indicating that stable overexpression of miR-210 was still effective after prolonged *in vivo* experiments (Supplementary Figure S2B, left panel). Of note, CDX2 and KRT20 (cytokeratin 20), two well-known differentiation markers [34, 35], were down-regulated in miR-210-overexpressing tumors compared to tumors derived from T20 control cells (Supplementary Figure S4A–S4B). Taken together, our results clearly indicate that miR-210 plays an important role in the hypoxia-induced colon TIC phenotype.

### Downregulation of miR-210 target gene *ISCU* leads to increased TIC self-renewal

**Figure 4:**
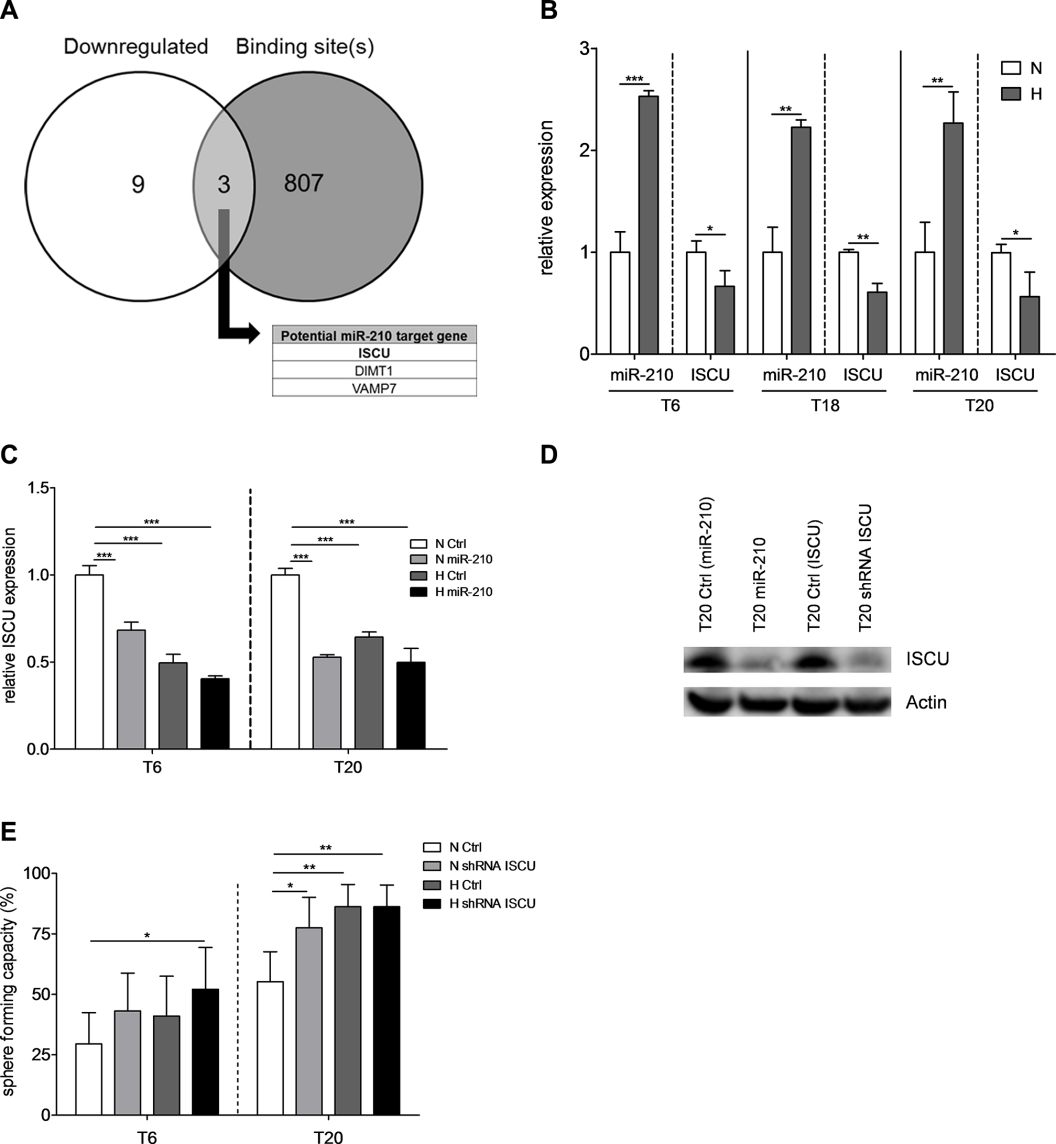
Downregulation of miR-210 target gene *ISCU* results in increased TIC self-renewal (**A**) Intersection between downregulated genes after stable overexpression of miR-210 in T20 SCs (white circle) and genes with at least one binding site for miR- 210- 3p in their 3′UTR (grey circle). Differentially expressed genes were determined by microarray experiments with T20 SCs after lentiviral transduction of either miR-210 or respective control vectors; significance cutoff was set at FDR < 0.05. Binding site information was retrieved from TargetScan (www.targetscan.org). (**B**) Relative expression of miR-210-3p and ISCU for SCs derived from primary tumors T6, T18 and T20, after 72 h of normoxic or hypoxic culture conditions, respectively. Data presented as mean ± SEM of three independent experiments. (**C**) Relative ISCU expression after lentiviral transduction of miR-210, after 72 h normoxia or hypoxia, respectively. Representative figure of 3 independent experiments; data are presented as mean ± SD. (**D**) ISCU protein expression in T20 cells after overexpression of miR-210 (lane 2) or shRNA-mediated knockdown of ISCU (lane 4), compared to respective controls (lanes 1 and 3); representative picture of 3 independent experiments. (**E**) Self-renewal capacity, determined with limiting dilution assays, after stable knockdown of ISCU in T6- and T20-derived SCs. Representative figure of 3 independent experiments; data presented as mean with 95% confidence interval. Statistical significance was assessed with unpaired Student's *t*-tests for (B) and (C) and with a Chi-square test for (E); *p < 0.05, **p < 0.01, and ***p < 0.001; N–normoxia, H–hypoxia, shRNA–short hairpin RNA.

In order to uncover the molecular mechanism underlying the hypoxia-induced TIC phenotype, we tried to identify potential target genes of miR-210, by performing microarrays for T20 SCs overexpressing miR-210. Among 12 significantly downregulated genes, 3 were reported to have a binding site for miR-210-3p in their three prime untranslated region (3′UTR) (Figure 4A, Supplementary Figure S5A). VAMP7 is known to play functional roles in cancer biology, as it is required for invadopodium formation and tumor cell invasion in diverse cancer types [36–38]. In our experimental setup, however, VAMP7 was downregulated and could thus not serve as an explanation for the increased tumorigenic behavior of TICs after overexpression of miR-210. Additionally, we couldn't observe any functional link between miR-210 and invasion (data not shown). DIMT1 has been linked to gastric inflammation and proliferation and was shown to be a direct miR-210 target gene [39]. In contrast to Kiga *et al.*, we couldn't see any effect of miR- 210 on cell proliferation in colon TICs (Supplementary Figure S3C–S3D). We therefore focused on the iron-sulfur cluster assembly protein ISCU, an accepted miR-210 target gene and known regulator of the metabolic response [40–45] which has a miR-210-3p binding site in its 3′UTR (Supplementary Figure S5B). As miR-210-induced repression of ISCU has been linked to altered metabolic activity in other cancer types [41,44], we hypothesized that this signaling axis might also be involved in the regulation of colon TIC activity. Indeed, hypoxia commonly led to the upregulation of miR-210 and to the repression of ISCU, pointing out an opposite expression pattern of both factors (Figure 4B), which was maintained in extracted T20 tumors following *in vivo* experiments (Supplementary Figure S2B). Furthermore, lentiviral transduction of miR-210 led to reduced ISCU levels, which were very similar to those of control cells under hypoxic conditions (Figure 4C). In agreement with others [41–45], our results nicely confirm that hypoxia-induced miR-210 negatively regulates ISCU expression, both on mRNA and protein level (Figure 4C–4D). Importantly, stable abrogation of ISCU (Figure 4D and supplementary Figure S2C for knockdown efficiency) allowed us to obtain a similar increase in sphere-formation capacity as overexpression of miR-210 (Figure 4E), without affecting cell proliferation, viability, or apoptotic rates (Supplementary Figure S3E– S3G) thus further highlighting the importance of the miR-210/ISCU signaling axis as a key regulator of TIC self-renewal activity.

### miR-210 and ISCU are relevant targets for CRC

**Figure 5:**
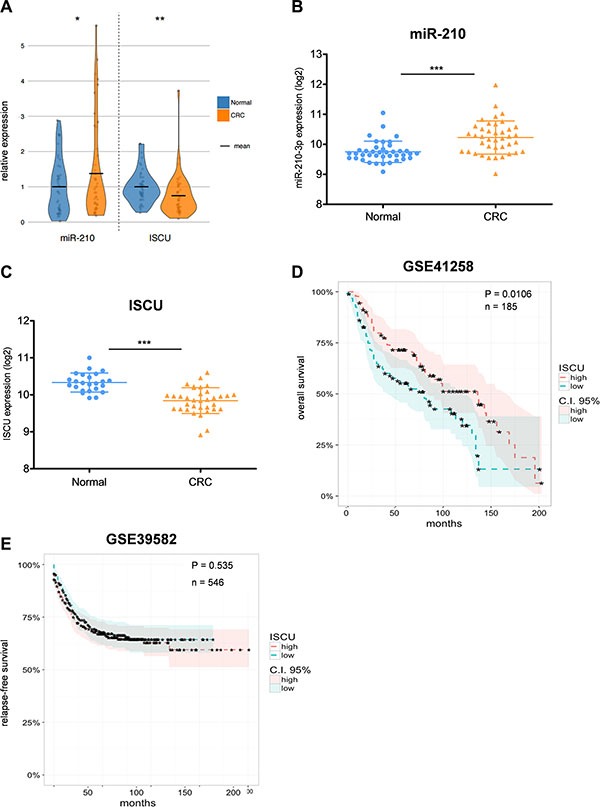
miR-210 and ISCU expression are clinically relevant for CRC patients (**A**) miR-210-3p and ISCU expression in 47 CRC tumor tissue samples relative to their matching normal counterparts; data are depicted as shaded dots overlaid with their respective distribution as violin plots, with means highlighted in black. (**B**) miR-210-3p expression in paired CRC and matching normal colon tissue samples of publicly available dataset GSE49246. Log_2_ expression intensities; data presented as mean ± SD. (**C**) ISCU expression in CRC and normal colon tissue samples in publicly available dataset GSE23878. Log_2_ expression intensities; data presented as mean ± SD. Kaplan-Meier curve showing overall (**D**) and relapse-free (**E**) CRC patient survival according to their ISCU expression level. Classification into “high” and “low” expression groups (with 95% confidence intervals) separated using their respective median ISCU expression value. Paired Student's *t*-test for (A) and (B), unpaired Student's *t*-test for (C), Cox proportional hazard model for (D) and (E); *p < 0.05, **p < 0.01, and ***p < 0.001.

miR-210 has recently been suggested as a potential prognostic marker in different epithelial cancers, including CRC [26, 27]. We therefore analyzed miR-210-3p expression in tumor tissue and normal colon counterparts of 47 CRC patients. Among different tested clinical factors, such as tumor size and stage or histological grade, we could observe a clear link between miR-210 and patient age, which is in line with previous studies [46], and which was also the case in normal colon samples, but only to a lesser extent (Supplementary Figure S6A). The correlation between age and miR-210 was more pronounced for women, entailing that female patients at an advanced age show the highest expression of miR- 210- 3p (Supplementary Figure S6B). In agreement with the study of Qu and colleagues [46], miR-210-3p levels were increased in CRC tumor samples compared to matching normal counterparts (Figure 5A), which was further validated in a publicly available dataset containing 40 paired stage II CRC and normal colon tissue samples (Figure 5B). Notably, in contrast to most other studies, we measured miR-210 and ISCU levels in the same patient cohort, which allowed us to observe that both factors show an opposite expression pattern in CRC (Figure 5A). While miR-210-3p was increased, ISCU gene expression was decreased in tumor tissues, which could be further confirmed in another dataset (Figure 5C). Of interest, HIF-1α gene expression was unchanged (HIF is rather expected to change on protein than on mRNA level), while hypoxia-responsive CA9 was highly increased in tumor samples (Supplementary Figure S7A–S7B), emphasizing the relevance of hypoxia-responsive factors in our CRC patient cohort. The prognostic value of ISCU was assessed in a public dataset containing overall survival data for more than 180 CRC patients. Indeed, low expression of ISCU thereby correlated with bad patient prognosis (Figure 5D). However, this trend could not always be confirmed in additional datasets (data not shown). Moreover, in dataset GSE39582 including more than 500 patients, disease-free survival, which is often regarded as a superior indicator for CRC patients' outcome compared to overall survival, did not correlate with reduced ISCU expression (Figure 5E). These results demonstrate that additional studies are needed to reach a conclusive state for the prognostic value of ISCU expression in CRC.

### miR-210/ISCU signaling axis regulates TIC self- renewal by inducing lactate production

**Figure 6:**
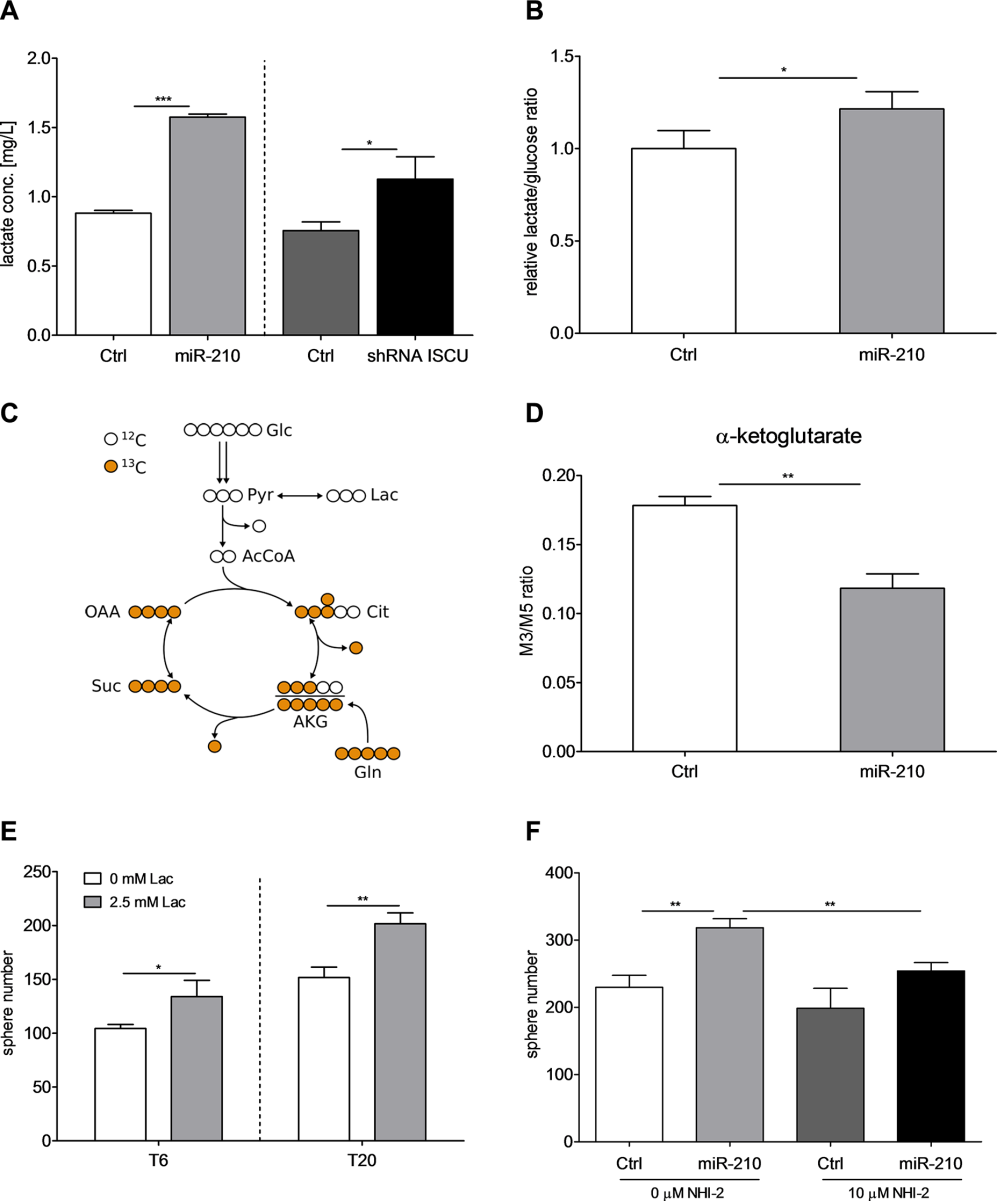
miR-210-induced lactate production enhances self-renewal capacity of colon TICs (**A**) Intracellular L-lactate levels after stable overexpression of miR-210 or knockdown of ISCU in T20-derived SCs. Representative figure of 2 independent experiments; data are presented as mean ± SD. (**B**) Ratio of produced lactate per consumed amount of glucose over 72 h for T20 miR-210-overexpressing and respective control cells. Representative experiment of 5 biological replicates, data presented as mean ± SD. (**C**) Schematic representation of central carbon metabolism. U-^13^C gln enters the TCA cycle as 5-times labeled (M5) α-ketoglutarate. One round in the TCA cycle leads to the generation of the M3 α-ketoglutarate isotopologue. (**D**) Ratio of M3 and M5 α-ketoglutarate isotopologues for miR-210-overexpressing T20 and corresponding control cells. Representative figure of 2 independent experiments; data presented as mean ± SD. (**E**) Sphere-forming capacity of T6 and T20 SCs, determined with 1000 cell assays, after stimulation with 2.5 mM L-lactate. Representative figure of 3 independent experiments; data presented as mean ± SD. (**F**) Sphere formation of T20 SCs after inhibition of LDHA with 10 μM NHI-2. Representative figure of 3 independent 1000 cell assays; data presented as mean ± SD. Statistical significance was assessed with unpaired Student's *t*-tests for (A), (B), (D), (E) and (F); ns – not significant, *p < 0.05, **p < 0.01, and ***p < 0.001; N–normoxia, H–hypoxia, Lac–lactate.

ISCU and miR-210 are known to influence the metabolism of cells by regulating mitochondrial activity [40, 41]. Accordingly, overexpression of miR-210 and downregulation of ISCU led to increased intracellular lactate levels (Figure 6A). In addition, both the ratio of produced lactate per consumed amount of glucose and the lactate secretion rate per cell were elevated after overexpression of miR-210 (Figure 6B and Supplementary Figure S8A), suggesting that the intracellular flux of glycolytic pyruvate was partially redirected from oxidation in the tricarboxylic acid (TCA) cycle to lactate production. To investigate how overexpression of miR-210 affects mitochondrial energy metabolism, we incubated the cells in the presence of a uniformly ^13^C-labeled glutamine (U-^13^C gln) tracer. After uptake by the cell, the carbon isotopes of the tracer are incorporated into downstream metabolites, thus generating distinct labeling patterns for each metabolite, which provides information on intracellular fluxes. U-^13^C gln thereby enters the TCA cycle as fully labeled (M5) α-ketoglutarate. One round of cycling in the TCA cycle leads to the formation of the M3 α-ketoglutarate isotopologue (Figure 6C). Interestingly, the M3/M5 ratio of α-ketoglutarate was decreased in miR- 210-overexpressing cells, indicating a decrease in TCA cycle activity (Figure 6D), which could also be confirmed in the α-ketoglutarate-derived metabolites proline and 2-hydroxyglutarate (Supplementary Figure S8B). These results are in line with the aforementioned rearrangement of pyruvate flux away from TCA cycle oxidation towards lactate production. To further understand if lactate could be involved in the miR-210-induced self-renewal of colon TICs, we stimulated TIC-enriched cultures with lactate. We could observe an increase in sphere formation after stimulation of T6 and T20 SCs with 2.5 mM lactate (Sigma-Aldrich) (Figure 6E). Most importantly, by disabling lactate production with the lactate dehydrogenase A (LDHA) inhibitor NHI-2 (Sigma-Aldrich), we were able to block out the promoting effects of increased miR-210 and repressed ISCU on self-renewal (Figure 6F and Supplementary Figure S8C). Taken together, these data nicely highlight that overexpression of miR-210, most probably via downregulation of its target gene *ISCU*, leads to reduced TCA cycle activity and enhanced lactate production, ultimately resulting in increased self-renewal of colon TICs.

## DISCUSSION

Recent data suggest that hypoxia is an important regulatory factor of TICs. Low oxygen tension leads to an increased TIC proportion in glioma patients [12, 15], drives gene expression of cancer cells towards a more immature phenotype [13], presses TIC-like cells derived from CRC cell lines into forming undifferentiated dense colonies [17], and induces reprogramming of non-tumorigenic cells towards a TIC-like behavior [14, 16]. Importantly, in the scope of this work, we show that hypoxia enhances the self-renewal capacity of both cell line- and patient-derived primary colon TIC cultures.

In recent years, a clear link between hypoxia and the expression of a number of HRMs has been established and several mechanisms have been proposed to describe the regulation of hypoxamiRs [21]: it has been speculated that hypoxia can induce a post-translational modification of Argonaute 2 that enhances its activity and thereby leads to a stronger incorporation of miRNAs into the RNA-induced silencing complex [47]. Other HRMs, among which miR- 210, are directly regulated by HIF-1α ([28–30] and Figure 2D) or are activated in a HIF-1α-dependent manner by other hypoxia-induced transcription factors (e.g. miR-10b which can be upregulated by HIF-1α-induced expression of TWIST [48]). The biological functions of hypoxamiRs have been extensively investigated and the role of miR-210 may be quite broad: besides affecting apoptosis, angiogenesis and cell cycle progression, miR-210 has been linked to the regulation of proliferation, tumor growth and mitochondrial metabolism [49]. Interestingly, miR-210 has also been linked to radioresistance and stemness of glioma TICs, reflected by a deregulated cell cycle and a decreased neurosphere formation after knockdown of miR-210 [50]. In the present study, we were able to show for the first time that stable overexpression of miR-210 results in increased sphere formation of colon TICs, thereby highlighting a clear link between hypoxia-induced miR-210 and TIC self-renewal activity.

**Figure 7:**
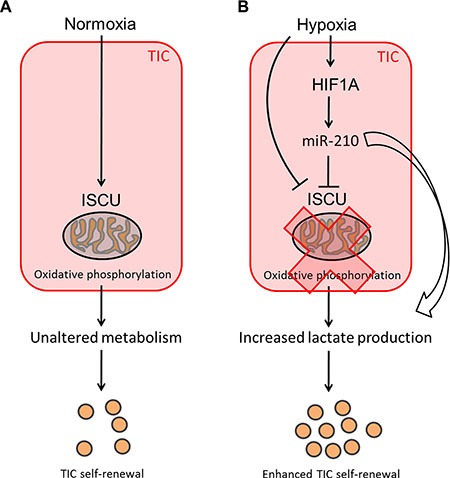
Potential mechanism leading to increased self-renewal of colon TICs under hypoxic conditions (**A**) Under normoxic conditions, ISCU and oxidative phosphorylation remain unaffected and TICs display a certain degree of self-renewal potential. (**B**) Hypoxia upregulates miR-210, which in turn represses ISCU, thereby leading to an altered TCA cycle activity. The resulting high L-lactate levels suggest that TICs display increased miR-210-induced glycolytic activity under hypoxic conditions, which might provide proliferation benefits and lead to enhanced self-renewal potential of colon TICs.

Solid tumors are composed of heterogeneous populations of cells, which can use different metabolic pathways to cover their needs in energy production. While the Warburg effect has been clearly demonstrated for most cancer types, the metabolic state of TICs remains controversial [51]. Although a subgroup of pancreatic cancer cells with TIC properties as well as glioma TICs are thought to be mainly dependent on mitochondrial respiration [52, 53], it was shown that TICs can readily switch to a glycolytic metabolism when oxidative phosphorylation is blocked [53]. Moreover, in many other cancer types, including melanoma [54], osteosarcoma [55], breast [56, 57], lung [58] and liver [59] cancer, TICs have been shown to preferentially display a glycolytic phenotype and decreased mitochondrial activity. In line with this, we could observe enhanced TIC self-renewal activity and increased lactate levels after stable overexpression of miR-210 or knockdown of its target gene *ISCU*, suggesting that reduced TCA cycle activity and lactate production correlate with colon TIC properties (Figure 7). Interestingly, ablation of the potential TIC marker CD44 has been shown to correlate with reduced glycolytic activity and increased chemosensitivity [60], further emphasizing a potential regulatory role of miR-210 in the metabolic reprogramming of colon TICs.

Hypoxia signaling has long been known to correlate with dismal cancer patient outcome [61] and it has recently been demonstrated that hypoxia-driven gene expression signatures have prognostic value for stage II and III CRC patients [62]. Thus, it seems important to decipher the complex cellular response to hypoxia and to determine the clinical relevance of individual hypoxia-responsive factors, such as miR-210 and ISCU. Indeed, miR-210 has been linked to poor patient prognosis for different epithelial cancer types, including CRC [26, 27]. In agreement with previous studies [46], we could show that miR-210 is upregulated in CRC tissue, compared to matching normal colon samples. Analyses of serum samples have shown that CRC patients display increased levels of circulating miR-210 compared to healthy controls [63,64], further emphasizing the diagnostic and prognostic value of miR-210. On the other hand, although the prognostic value of ISCU has been proven for breast cancer as well as for head and neck squamous cell carcinoma [44], our results of the correlation between ISCU expression and patient survival show that further studies are needed to establish a conclusive state for CRC. It seems reasonable that combined biomarker panels comprising both miRNA and mRNA data are able to outperform one-level biomarkers. We might thus speculate that the combined expression of hypoxia-induced miR-210 and its target gene *ISCU* may be preferentially used for prognostic assessment of CRC patients.

Recently, lactate metabolism and anti-cancer treatments targeting lactate shuttles have gained a lot of interest in the scientific community [65]. Emerging data suggests that tumor cells can shuttle lactate to neighboring cancer cells, adjacent stroma, and vascular endothelial cells and that lactate is able to promote tumor inflammation and angiogenesis [66, 67]. Furthermore, it has been shown that lactate also regulates TIC activity by promoting a stem-like and aggressive phenotype in breast cancer [68] and by enhancing self-renewal capacity of liver TICs [59]. Moreover, blocking lactate production by specifically targeting LDHA leads to differentiation of glioblastoma TICs [69] and to reduced lung cancer tumorigenesis [58]. Along these lines, increased lactate levels, both via direct stimulation or indirectly via overexpression of miR-210 or knockdown of ISCU, resulted in enhanced self-renewal capacity of our colon TIC cultures. Importantly, the promoting effect of miR-210 on TIC self-renewal could be reversed by blocking lactate production, highlighting again the central role of this miRNA in the regulation of hypoxia-induced TIC activity.

Taken together, our study underscores the role of miR-210/ISCU signaling in regulating cancer cell metabolism, lactate production and self-renewal activity of TICs as well as its clinical relevance for CRC patients. Overall, we were able to show and confirm that miR- 210 is a key mediator of the hypoxic response, which ultimately drives tumor initiation by regulating the self-renewal capacity of colon TICs.

## MATERIALS AND METHODS

### Patients

Human colon tissue samples were collected by the Integrated Biobank of Luxembourg (IBBL, www.ibbl.lu) in accordance with institutional guidelines, following the standard pre-analytical code for biospecimens by the IBBL [70], as previously described [71]. All human samples used in the scope of this work were donated freely and written informed consent as well as ethical approval from the Comité National d'Ethique de Recherche du Luxembourg (Reference 201009/09) and from the Ethics Review Panel of the University of Luxembourg (ERP-16-032) were obtained. Primary CRC tumor as well as matched non-neoplastic colon tissue were collected from 17 female and 30 male patients with an average age of 67.2 years (ranging from 47 to 88), featuring adenoma (*n* = 3), stage I (*n* = 7), stage II (*n* = 18), stage III (*n* = 17), and stage IV (*n* = 2) CRC patients.

### Cell culture

HCT116, HT29 and LS174t cell lines were obtained from the American Type Culture Collection (Rockville, USA), recently authenticated, and maintained in recommended culture conditions. Spheroid cultures were established both from CRC cell lines and from fresh primary colon cancer tissue (T6, T18, and T20) immediately after surgical resection. All SCs used within this study were established, enriched for TICs and fully characterized as previously described [8]. For hypoxic conditions, cells were maintained at 1% O_2_.

### Viral transductions

Ready-to-use lentiviral particles were used to generate SCs with 1) stable overexpression of miR-210 (Biosettia), 2) stable knockdown of either ISCU or HIF-1α via short hairpin RNA (Santa Cruz), or 3) respective control vectors. Cells were transduced at a multiplicity of infection of 3-5 and selected with puromycin. Transduction efficiency was checked via microscope and qPCR.

### Sphere and colony formation assays

SCs were dissociated into single cells and sphere formation assays were performed at different densities, as previously described [8]. Single cell assay: cells were seeded at a density of 1 cell per well and, after 10 days of culture, resulting spheroids were counted and measured under a microscope. Only wells that initially contained one single cell were retained and two 96-well plates were counted for each condition. Limiting dilution assay: different cell densities were used to assess TIC frequency after 10 days using the extreme limiting dilution analysis (ELDA) software [72]. 1000 cell assay: 1000 cells/well were seeded in ultra-low-attachment (ULA) 6-well plates and resulting spheres were counted after 7 days. Colony formation assay: clonogenic capacity was tested by seeding 250 cells per well; after 10 days of culture in serum-containing medium, resulting colonies were stained and counted under a microscope.

### *In vivo* tumor formation assays

NOD scid gamma (NOD.Cg-Prkdc^scid^ Il2rg^tm1Wjl^/SzJ) (NSG) mice were bred in-house and experiments performed according to all applicable laws and regulations subsequent to approval by the institution's animal care and ethical committee (Permit Number: 14-MDM-02). Based on prior *in vivo* characterization of our SCs [8], different cell doses (i.e. 10,000 or 5 cells per injection) were selected and resuspended in 100 μL of 10.8 mg/mL growth factor-reduced Matrigel (BD Biosciences) and subcutaneously injected in the flank of 6-week-old mice and tumor growth was calculated with the formula V = (π/6 × L × W × H). Mice were sacrificed after 1 month (for injections of 10,000 cells) or 10 weeks (for injections of 5 cells) respectively, tumor incidence as well as weight of resected tumors were assessed, and miR-210-3p expression levels were determined by qPCR.

### Proliferation rates

Growth curves of different SCs were established by plating 100,000 cells/well in ULA 6-well plates. Resulting spheroids were dissociated after 24 h, 48 h or 72 h respectively and cells were counted on a Cedex XS (Roche) cell counter.

### Caspase 3 activity assay

Apoptotic rates of different SCs were determined with a caspase 3 activity test, as previously described [73]. Briefly, cleavage of specific caspase substrate Ac-DEVD-AFC (Biomol) was monitored by measuring changes in fluorescence intensity (excitation wavelength 400 nm, emission wavelength 505 nm) on a CLARIOstar microplate reader (BMG labtech) to determine the extent of apoptosis.

### Lactate assay

Three days after seeding, SCs were dissociated and 1.5 million cells per condition were used to assess intracellular L-lactate levels with a colorimetric L-lactate assay kit (Abcam), following the user's manual.

### Extracellular lactate and glucose levels

Extracellular lactate and glucose concentrations were quantified with a Yellow Springs 2950D analyzer (Yellow Springs Instruments Inc.), following standard protocols. Briefly, supernatants of 150,000 T20 miR-210-overexpressing or respective control cells were filtered (PHENEX-RC 4 mm, 0.2 μm; Phenomenex) and glucose and lactate levels were quantified by using external calibration standards. Glucose uptake and lactate secretion rates were calculated by normalizing concentrations to the respective cell numbers of each condition.

### Analysis of glutamine metabolism

Stable miR-210-overexpressing and respective control T20 SCs were dissociated and 150,000 single cells per well were incubated with U-^13^C gln tracer. Cells were collected after 72 h and metabolites were extracted and analyzed by gas chromatography-mass spectrometry, as previously described [74].

### Flow cytometry

CD44 surface marker expression was determined by flow cytometry. Samples were prepared as previously described [5] and analyzed on a FACS Canto II. CD44 PE-Cy7 and PE-Cy7 Mouse IgG2b, κ (BD Biosciences) were used as primary antibody and isotype control, respectively. Cell viability was assessed by staining dead cells with 1 μM Sytox blue (Life technologies).

### Cell lysis and Western blot analysis

Cell lysis was performed at 4°C, using ice-cold buffers. After a washing step with 1x PBS, adherent T20 cells were lyzed on the dish with RIPA buffer (Thermo Fisher) containing 1% SDS. After addition of 1x Lämmli buffer, lysates were vortexed and supernatants were heated at 95°C for 5 minutes. Proteins were subjected to SDS-PAGE (12% gels) and probed with the respective antibodies. Primary antibodies against ISCU 1/2 (SC- 28860) and actin (MAB1501) were purchased from Santa Cruz or Merck Millipore, respectively. Signals were detected on a Fusion FX (Vilber Lourmat) imaging platform, using an ECL solution containing 2.5 mM luminol, 100 mM Tris/HCl pH 8.8, 0.2 mM para-coumaric acid, and 2.6 mM hydrogenperoxide [75].

### Real-time qPCR

Total RNA, both from patient biopsies (in collaboration with the IBBL) and from SCs, was extracted using the miRNeasy Mini Kit (Qiagen) and reverse transcription was performed with the miScript II RT Kit (Qiagen) according to the user's manual. qPCR reactions were performed on 7500 Fast Real-Time PCR systems (Applied Biosystems) using Absolute Blue qPCR SYBR Green Low ROX (Thermo Scientific) and miScript Primer Assays (Qiagen) for miRNAs or specific primer pairs (Supplementary Table S2) for genes of interest. Cycles of 40 × (95°C for 15 sec., 60°C for 30 sec.) or 40 × (95°C for 30 sec., 60°C for 30 sec. and 72°C for 30 sec.) were used for miRNAs and mRNAs, respectively. Expression levels of genes and miRNAs of interest were normalized against the geometric mean of multiple reference targets using qbase^+^ (Biogazelle), according to the MIQE guidelines [76].

### mRNA and miRNA expression arrays

Gene expression profiling experiments were performed and analyzed as previously described [8]. A false discovery rate (FDR) < 0.05 was set as significance cutoff value for the determination of differentially expressed genes. Microarray data are available in the GEO database under the accession number GSE80236.

miRNA expression was assessed with SmartChip Human microRNA Panel v3 arrays (WaferGen Biosystems). RNA quality control was performed on 2100 Bioanalyzer instruments (Agilent). Expression values were normalized in qbase+ (Biogazelle) using the *global mean* approach, as recommended for large screening studies. Linear modelling with an empirical Bayesian approach was applied with the Bioconductor *limma* R package [77]. Hypoxia-responsive miRNAs were identified by setting a *p*-value < 0.05 as significance threshold for differential expression.

### Public datasets

Public dataset GSE49246 [78] was used to compare miR-210-3p expression in 40 stage II CRC and paired normal colon tissue samples. ISCU gene expression was retrieved from GSE23878 [79], a public microarray dataset with 35 CRC and 24 non-cancerous colorectal tissue samples.

The effect of ISCU expression on overall and relapse-free patient survival was assessed in datasets GSE41258 [80] and GSE39582 [81], respectively. Median values were used to separate CRC patients into “high” and “low” groups, according to their ISCU gene expression level. Kaplan-Meier curves were obtained with the *survival* [82] R package and drawn with the *ggplot2* [83] and *ggfortify* [84] R packages.

### Data analysis

GraphPad Prism version 5 software and R 3.2 [85] were used for statistical analysis. We used Student's *t*-tests to compare two conditions and 2-way ANOVA to compare *in vivo* tumor growth between groups. The ELDA [72] software and Chi-square tests were used for the analysis of limiting dilution assays. For Kaplan-Meier plots, statistical significance was assessed with the Cox proportional hazard model (function *coxph* from the *survival* R package) and plots were obtained using the *autoplot* function from the *ggfortify* R package. Statistical significance of patients' age and gender information was assessed with linear regressions (function *lm*) in R. All experiments were performed in at least three independent biological replicates and data is reported as mean ± SD, unless otherwise stated.

## SUPPLEMENTARY MATERIALS FIGURES AND TABLES


