# Supplementary material for: Hypoxia-responsive miR-210 promotes self-renewal capacity of colon tumor-initiating cells by repressing ISCU and by inducing lactate production
Source: Oncotarget. 2016 Aug 31;7(40):65454–70. doi: 10.18632/oncotarget.11772 (PMC5323168; doi:10.18632/oncotarget.11772)
Supplement: Supplementary file 1 [file oncotarget-07-65454-s001.pdf]

Hypoxia-responsive miR-210 promotes self-renewal capacity of colon tumor-initiating cells by repressing ISCU and by inducing lactate production

Supplementary Materials

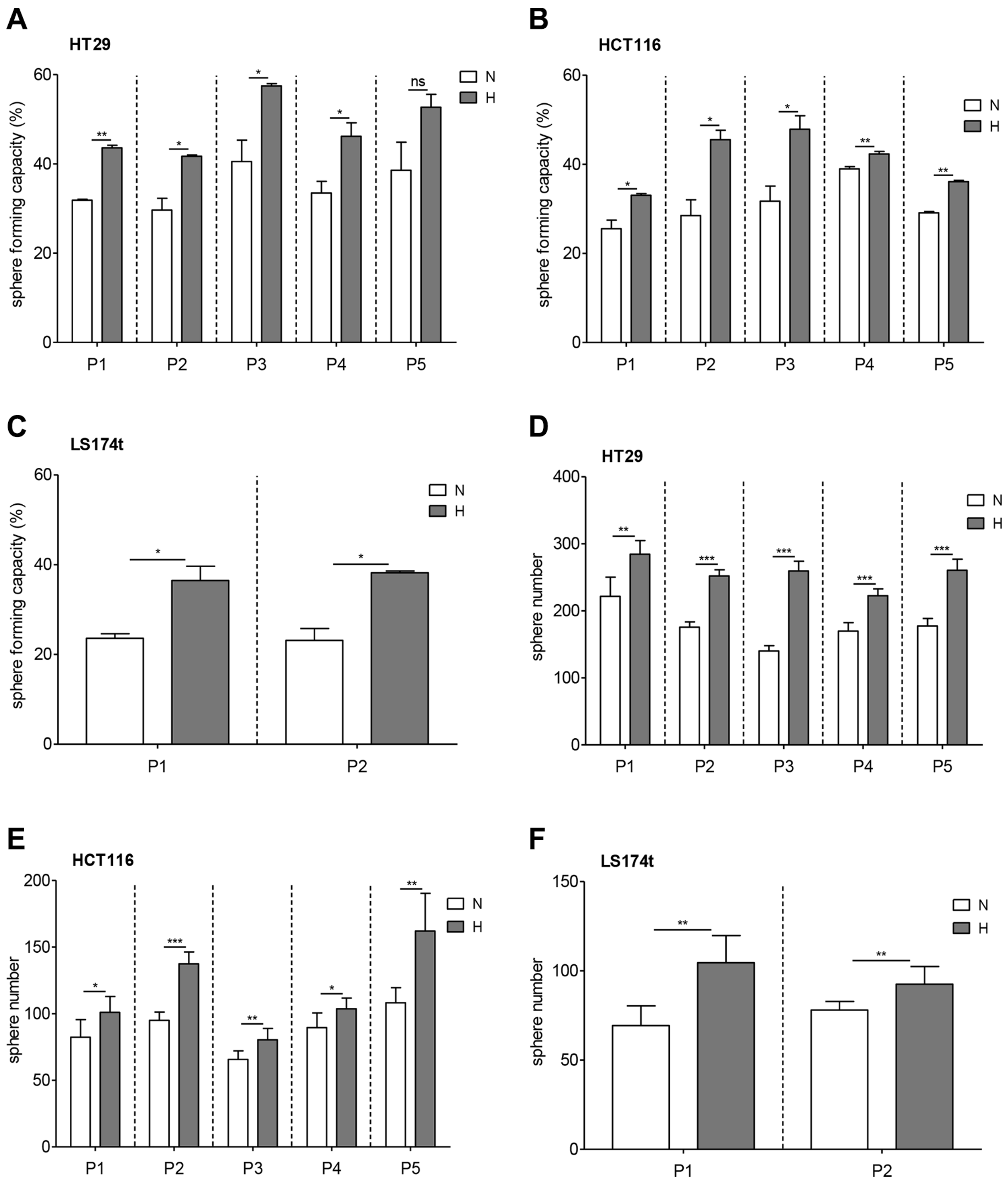

Supplementary Figure S1: Hypoxia promotes colon TIC self-renewal over consecutive passages. Single cell assays were performed for (**A**) HT29, (**B**) HCT116, and (**C**) LS174t SCs over consecutive passages under normoxic and hypoxic conditions, respectively. 1,000 cell assays were performed for (**D**) HT29, (**E**) HCT116, and (**F**) LS174t SCs over consecutive passages under normoxic and hypoxic conditions, respectively. Data are presented as mean ± SD; unpaired Student's *t*-test was used to compare normoxia and hypoxia; ns = not significant, *$p < 0.05$, **$p < 0.01$ and ***$p < 0.001$; N–normoxia, H–hypoxia.

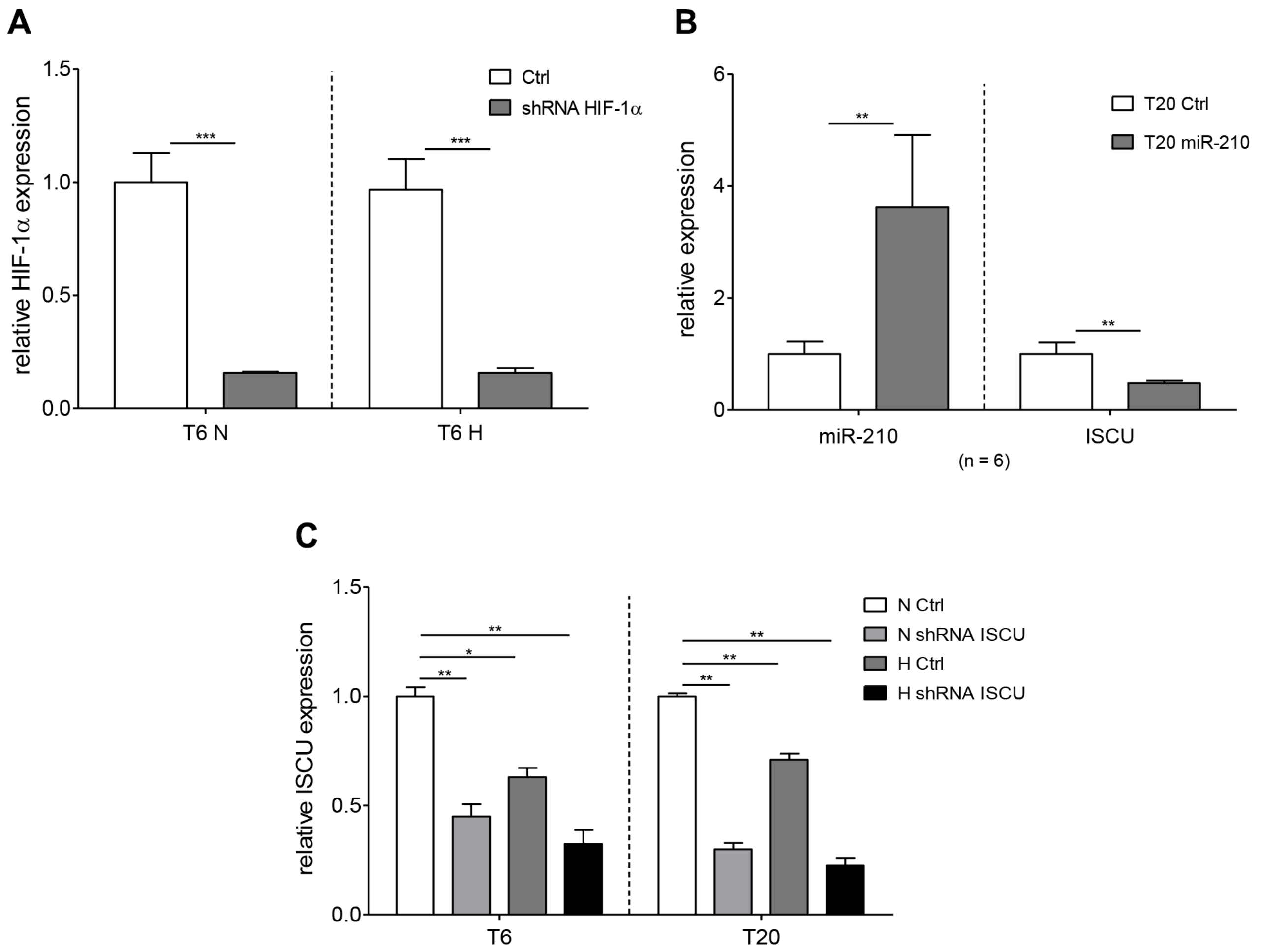

Supplementary Figure S2: Knockdown and overexpression controls. (**A**) Evaluation of HIF-1α knockdown after stable transduction of lentiviral particles with HIF-1α short hairpin RNA and after 72 h under normoxic or hypoxic conditions, respectively. Representative figure of 2 independent experiments with technical triplicates. (**B**) Relative expression of miR-210-3p and ISCU in extracted T20 tumors, 1 month after subcutaneous injection of 10,000 miR-210-overexpressing or control cells, respectively; $n = 6$ mice/group. (**C**) Relative expression of ISCU in T6 and T20 SCs after lentiviral transduction of ISCU short hairpin RNA or control vector, respectively. SCs were exposed to 72 h normoxic or hypoxic conditions prior to assessing ISCU expression levels. Representative figure of 2 independent experiments with technical duplicates; data represented as mean ± SD; unpaired Student's *t*-tests were used for (A) and (C); paired Student's *t*-test was used for (B); *$p < 0.05$, **$p < 0.01$, and ***$p < 0.001$; N–normoxia, H–hypoxia, shRNA–short hairpin RNA.

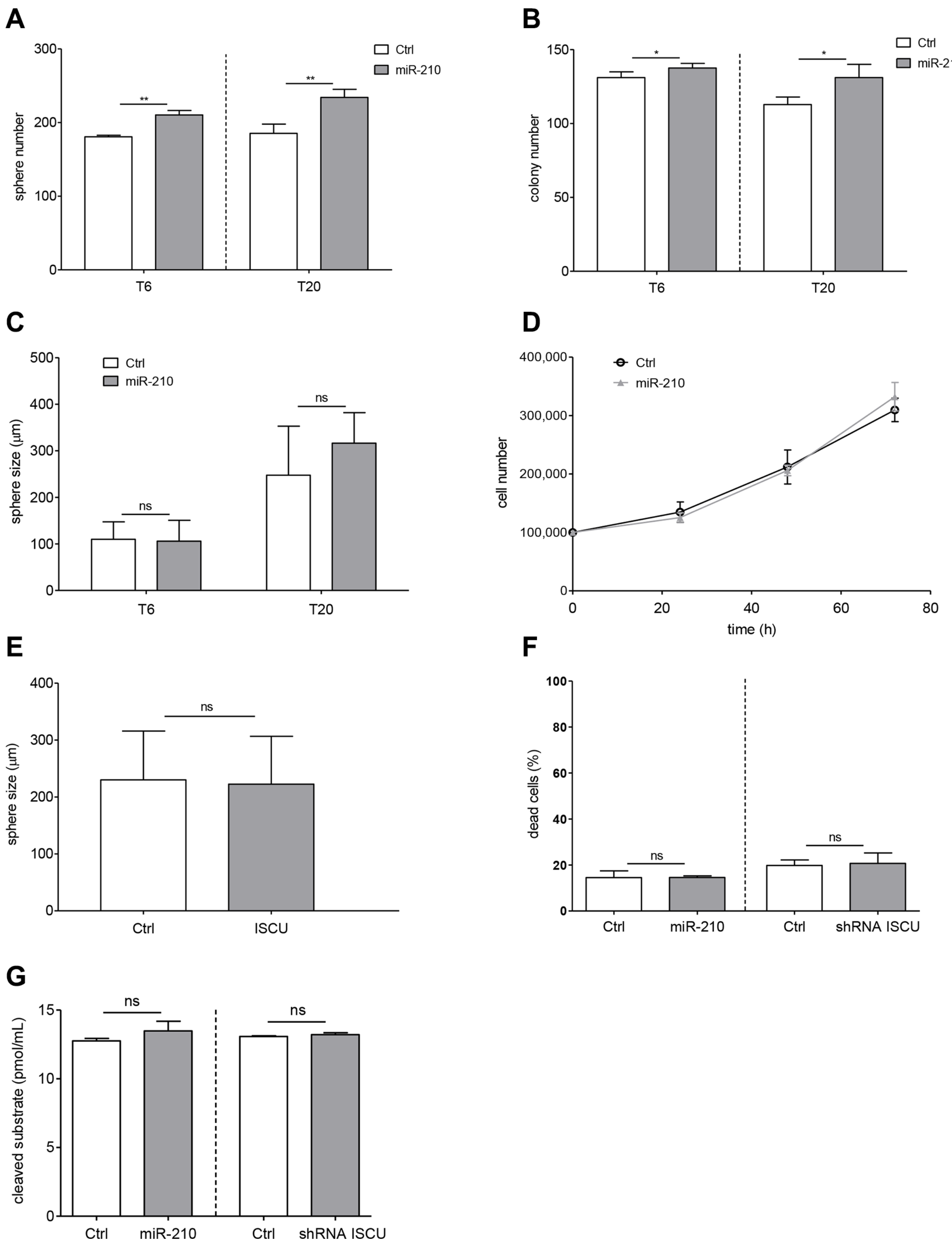

Supplementary Figure S3: miR-210 promotes sphere and colony formation without affecting proliferation, viability, or apoptotic rates of colon TICs. (**A**) Sphere numbers in T6 and T20 1,000 cell assays after stable overexpression of miR-210. (**B**) Colony formation assays were used to determine the clonogenic capacity of T6 and T20 SCs after stable overexpression of miR-210. (**C**) Size of single-cell-derived miR-210-overexpressing or control T6 and T20 spheroids, after 10 days in culture. (**D**) Growth curve of miR-210-overexpressing and control T20 SCs over 3 days. (**E**) Size of single-cell-derived T20 control spheroids and T20 SCs after lentiviral transduction of ISCU short hairpin RNA, after 10 days in culture. (**F**) Assessment of dead T20 cells by flow cytometry after stable overexpression of miR-210 or knockdown of ISCU, respectively. (**G**) Apoptotic rates, assessed by caspase 3 activity assay, after stable overexpression of miR-210 or knockdown of ISCU in T20 SCs. Results are representative of at least 2 independent experiments with 3 technical replicates for (A), 4 for (B), 8 for (C), 3 for (D), 8 for (E), 2 for (F) and 3 for (G). Data are presented as mean ± SD; unpaired Student's *t*-tests were used to compare two groups; ns = not significant, $*p < 0.05$, and $**p < 0.01$.

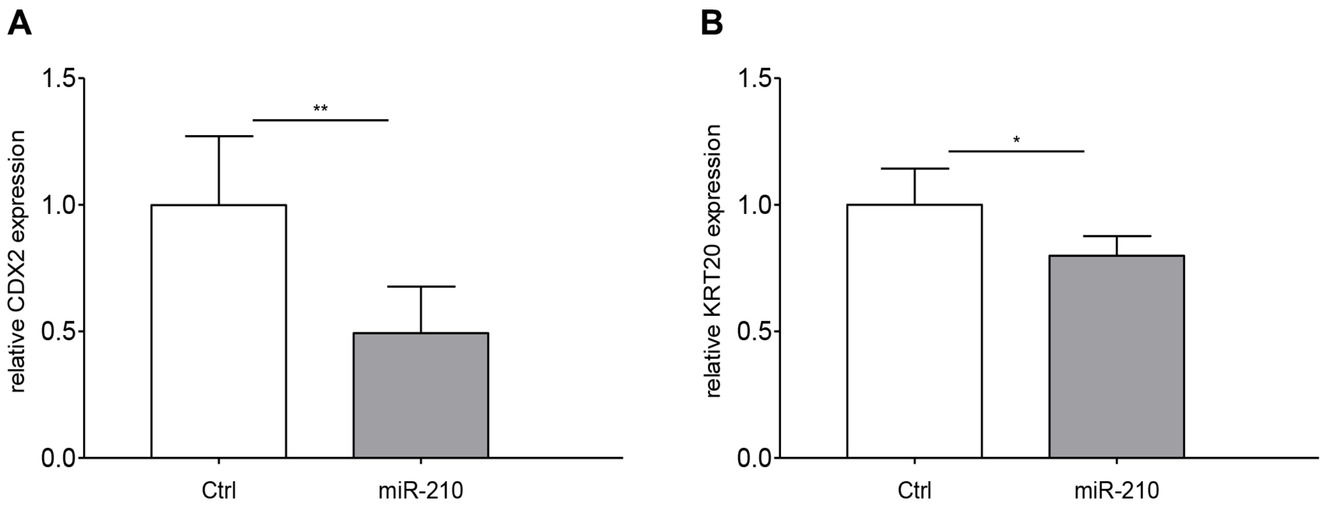

Supplementary Figure S4: Decreased expression of differentiation markers in xenografts after overexpression of miR-210. Relative expression of (**A**) CDX2 and (**B**) KRT20 (cytokeratin 20) in extracted T20 tumors, following subcutaneous injection of 10,000 cells with stable overexpression of miR-210 or respective control vector. Data are represented as mean ± SD; $n = 6$ mice/group; unpaired Student's t-test was used to assess statistical significance; *$p < 0.05$ and **$p < 0.01$.

A

Gene	log2 fold change	adj. P-value	miR-210 binding site
BAAT	-0,82	0,0019	no
VAMP7	-0,66	0,0046	yes
MAP2K6	-0,63	0,0475	no
PLXND1	-0,62	0,0015	no
DIMT1	-0,54	0,0055	yes
ISCU	-0,54	0,0497	yes
SLC2A2	-0,53	0,0133	no
QPRT	-0,50	0,0319	no
NUPR1	-0,47	0,0319	no
NPC1L1	-0,43	0,0319	no
TEX9	-0,42	0,0319	no
RAB8B	-0,39	0,0392	no

Downregulated genes after overexpression of miR-210 in T20 SCs. Differentially expressed genes (DEGs) were determined by setting a false discovery rate < 0.05. DEGs are ranked according to the absolute value of $\log_2$ fold change (expression of miR-210 vs. Ctrl).

B

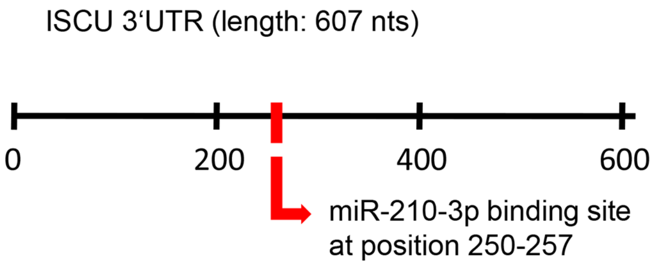

Supplementary Figure S5: *ISCU* is a potential miR-210 target gene. (**A**) List of 12 miR-210-responsive down-regulated genes, identified with microarray experiments for T20 SCs after lentiviral transduction of either miR-210 or respective control vectors; significance cutoff was set at FDR < 0.05. (**B**) Schematic representation of the miR-210-3p binding site in the 3′UTR of its potential target gene *ISCU*.

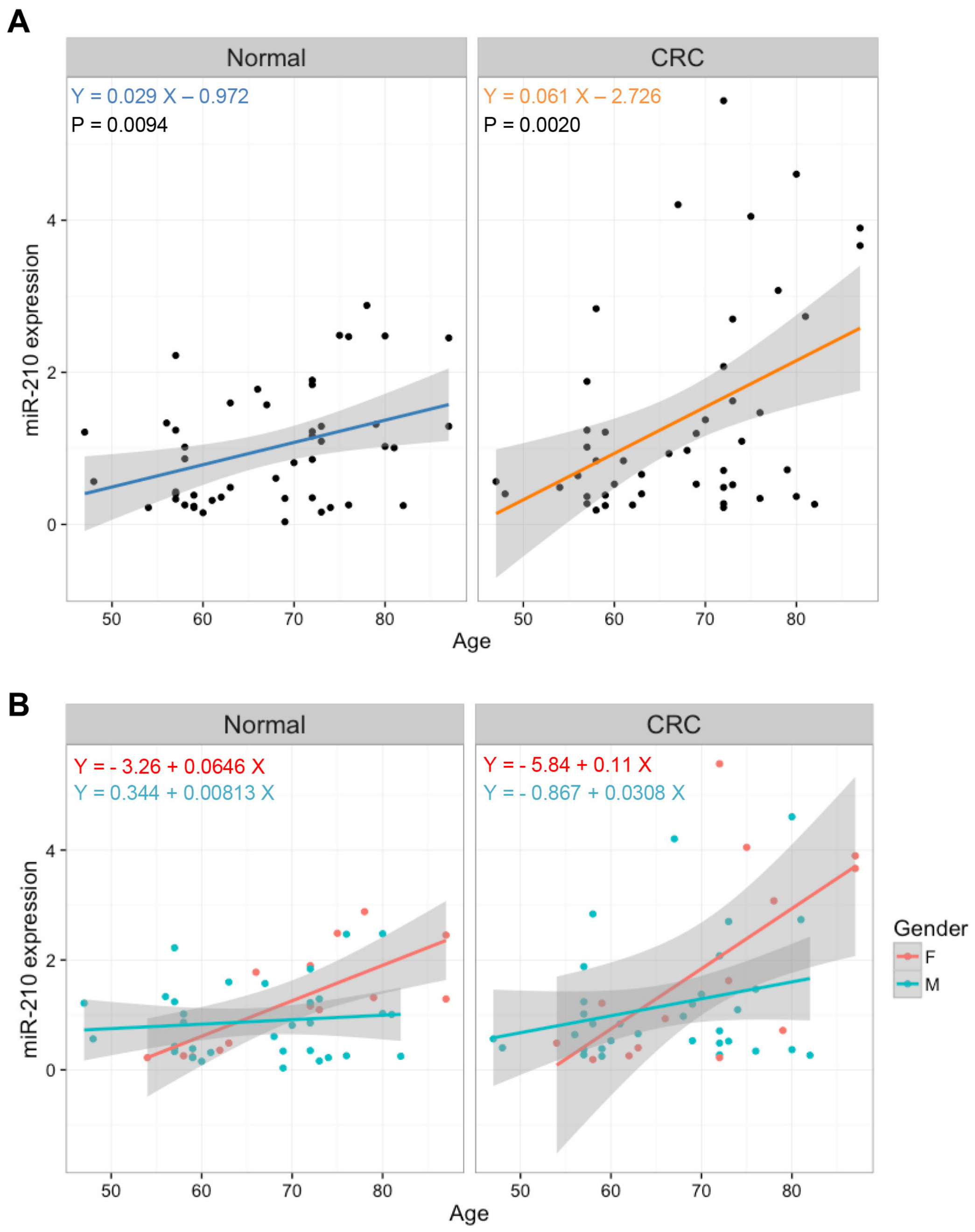

Supplementary Figure S6: miR-210-3p expression correlates with patients' age and gender. (**A**) Linear regression of the expression of miR-210-3p explained by patients' age in normal counterparts (left panel) and corresponding CRC samples (right panel); $n = 47$; grey areas indicate 95% confidence interval. (**B**) Multiple linear regressions of the expression of miR-210-3p explained by patients' age and gender. Models built for both normal colon (left) and paired CRC samples (right); $*p < 0.05$ for gender vs. age interaction in both cases; grey areas indicate 95% confidence interval.

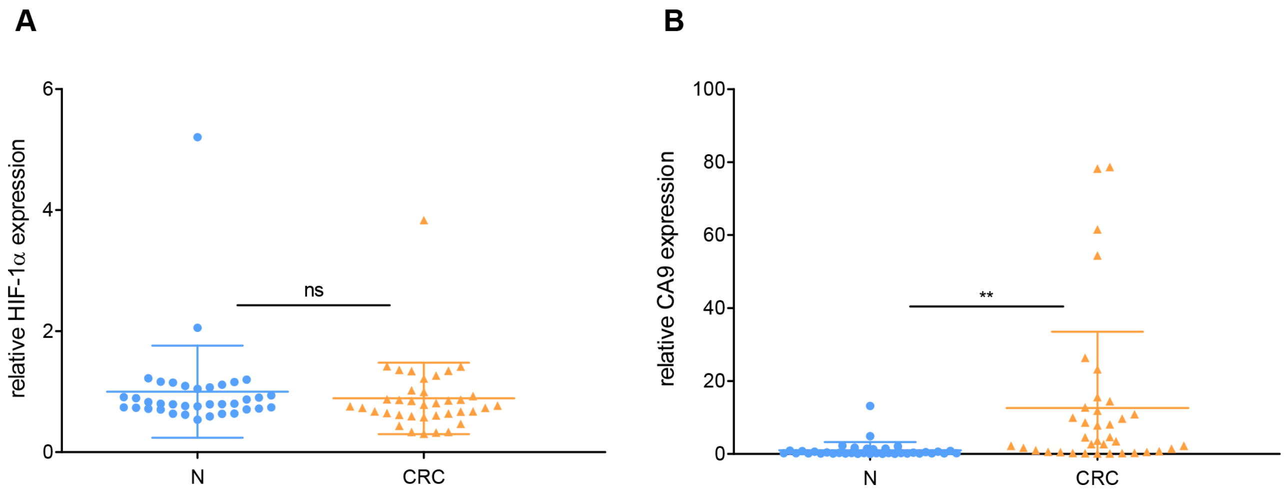

Supplementary Figure S7: HIF-1α and CA9 expression in CRC patient samples. Relative expression of (**A**) HIF-1α and (**B**) CA9 in 47 CRC patient tumor samples, compared to their matching normal colon tissue. Paired Student's *t*-test was used to compare both groups; ns = not significant; ***$p < 0.01$.

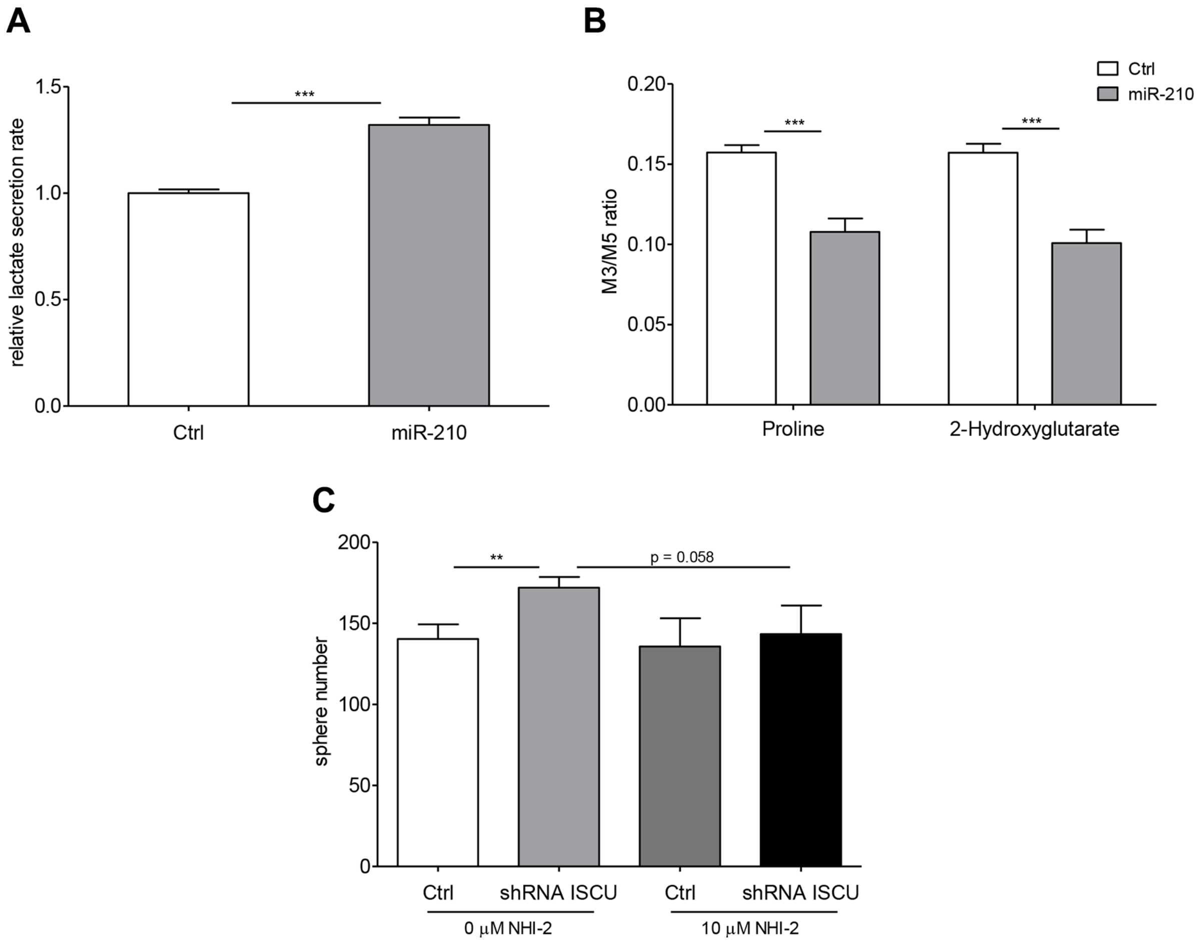

Supplementary Figure S8: Overexpression of miR-210 and knockdown of ISCU result in altered TCA cycle activity. (**A**) Relative extracellular lactate levels in T20 miR-210-overexpressing and respective control SCs, normalized to the corresponding cell numbers after 72 h. Data presented as mean ± SD; representative experiment of 5 biological replicates with technical triplicates. (**B**) M3/ M5 isotopologue ratios of proline and 2-hydroxyglutarate, for T20 miR-210-overexpressing or control SCs, after 72 h of incubation with U-13C gln. Data presented as mean ± SD; representative figure of 2 independent experiments, each with technical triplicates. (**C**) Sphere formation of T20 SCs after stable knockdown of ISCU and inhibition of LDHA with 10 μM NHI-2. Representative figure of 3 independent 1,000 cell assays with technical triplicates; data presented as mean ± SD. Unpaired Student's *t*-tests were used to compare two groups; ***$p < 0.01$; ****$p < 0.001$.

Supplementary Table S1: Hypoxia-responsive miRNAs in HT29 and HCT116 SCs

HT29

name	log fold change	fold change	*p*-value
miR-210-3p	0.57	3.70	1.82E-03
miR-600	–0.42	0.39	1.42E-03
miR-885-5P	0.41	2.55	2.84E-03
miR-1539	0.33	2.12	1.57E-02
miR-708-3p	–0.32	0.48	1.96E-03
miR-95-3p	–0.31	0.49	2.13E-02
miR-491-5P	0.31	2.04	1.61E-03
miR-558	0.29	1.94	7.05E-03
miR-218-5p	0.29	1.94	4.61E-03
miR-3142	0.28	1.88	4.12E-03
miR-380-3p	0.25	1.79	1.48E-02
miR-875-3P	–0.25	0.56	5.40E-03
miR-148a-3p	0.22	1.66	3.55E-02
miR-155-5p	0.22	1.66	1.87E-02
miR-576-5P	0.18	1.53	3.16E-02
miR-574-3P	–0.18	0.66	1.59E-02
miR-520g-3p	–0.18	0.66	2.40E-02
miR-582-5P	–0.18	0.66	1.60E-02
miR-671-3P	0.18	1.50	4.73E-02
miR-935	–0.18	0.67	4.11E-02
miR-21-3p	0.17	1.48	2.68E-02
miR-200a-5p	0.16	1.46	3.17E-02
miR-3922	–0.13	0.74	4.47E-02

HCT116

name	log fold change	fold change	*p*-value
miR-210-3p	0.61	4.07	3.90E-05
miR-301b	0.38	2.41	2.73E-02
miR-30c-2-3p	–0.34	0.45	3.01E-03
miR-148b-5p	0.33	2.16	2.75E-03
miR-520e	–0.33	0.46	3.30E-03
miR-34c-3p	–0.31	0.49	1.53E-02
miR-3157-5p	–0.30	0.50	2.42E-02
miR-520f-3p	–0.29	0.52	4.47E-02
miR-24-1-5p	0.29	1.94	2.85E-02
miR-449c-5p	0.26	1.81	1.65E-02
miR-4318	0.25	1.80	2.36E-02
miR-875-3p	–0.23	0.58	1.28E-02
miR-181d-5p	0.23	1.71	1.35E-02
miR-558	–0.20	0.62	3.42E-02
miR-891A	–0.19	0.64	4.59E-02
miR-181b-5p	0.19	1.55	2.75E-02

Differentially expressed miRNAs (DEMs) were determined by setting a p-value < 0.05 as significance threshold. DEMs are ranked according to the absolute value of $\log_{10}$ fold change (expression under hypoxia vs. normoxia).

Supplementary Table S2: RT-qPCR primer pairs used in this study

Gene symbol	Forward	Reverse
EEF1A1	5'-TTG-TCG-TCA-TTG-GAC-ACG-TAG-3'	5'-TGC-CAC-CGC-ATT-TAT-AGA-TCA-G-3'
ACTB	5'-GCA-AAG-ACC-TGT-ACG-CCA-ACA-3'	5'-ACA-CGG-AGT-ACT-TGC-GCT-CAG-3'
YWHAZ	5'-ACT-TTT-GGT-ACA-TTG-TGG-CTT-CAA-3'	5'-CCG-CCA-GGA-CAA-ACC-AGT-AT-3'
ISCU	5'-TCT-GCC-TTC-CTC-CCG-TGA-AAC-T-3'	5'-GAG-GGC-TCA-TTT-CTT-CTC-TGC-C-3'
HIF1A	5'-CGT-TCC-TTC-GAT-CAG-TTG-TC-3'	5'-TCA-GTG-GTG-GCA-GTG-GTA-GT-3'
CDX2	5'-ACA-GTC-GCT-ACA-TCA-CCA-TCC-G-3'	5'-CCT-CTC-CTT-TGC-TCT-GCG-GTT-C-3'
KRT20	5'-TGT-CCT-GCA-AAT-TGA-TAA-TGC-T-3'	5'-AGA-CGT-ATT-CCT-CTC-TCA-CTC-TCA-TA-3'
CA9	commercial RT^2 qPCR primer assays for human Ca9 (Qiagen)	
